# Visual outcomes and patient satisfaction after bilateral implantation of an enhanced monofocal intraocular lens: a single-masked prospective randomized study

**DOI:** 10.1007/s10792-024-02946-9

**Published:** 2024-02-26

**Authors:** Rosa Giglio, Alex Lucia Vinciguerra, Marianna Presotto, Kamil Jonak, Robert Rejdak, Mario Damiano Toro, Mayank Ambarish Nanavaty, Daniele Tognetto

**Affiliations:** 1 Department of Medicine, Surgery and Health Sciences, University of Trieste, Ospedale Maggiore, Piazza Ospedale 1, 34129 Trieste, Friuli-Venezia Giulia Italy; 2 Department of Biomedical Engineering, Lublin University of Technology, 20-618 Lublin, Poland; 3 Department of Psychiatry, Psychotherapy and Early Intervention, Medical University of Lublin, 20-093 Lublin, Poland; 4 Department of General and Pediatric Ophthalmology, Medical University of Lublin, 20-093 Lublin, Poland; 5 Eye Clinic, Public Health Department, University of Naples Federico II, 80138 Naples, Italy; 6 Sussex Eye Hospital, University Hospitals Brighton NHS Foundation Trust, Brighton, UK

**Keywords:** Cataract surgery, Enhanced monofocal IOL, Intermediate vision, Spectacle independence

## Abstract

**Purpose:**

To evaluate and compare the visual outcomes of an enhanced monofocal intraocular lens (IOL) with two different monofocal IOLs.

**Setting:**

Eye Clinic, Department of Medicine, Surgery and Health Sciences, University of Trieste, Trieste, Italy.

**Design:**

Prospective, single-center, single-masked, randomized controlled clinical study.

**Methods:**

The study included patients undergoing phacoemulsification and IOL implantation. Patients were consecutively randomized by block randomization and assigned in a 1:1:1 allocation ratio to three study arms to bilaterally receive Tecnis Eyhance™ (model ICB00) or Tecnis^®^ monofocal 1-piece (model PCB00) or Clareon^®^ monofocal (model CNA0T0), respectively. Monocular and binocular (both corrected and uncorrected) visual acuities for far, intermediate and near were registered and compared among groups at 3 months. To track changes in patient quality of life, the Catquest-9SF questionnaire was administered to each patient before and after cataract extraction.

**Results:**

Ninety patients (30 for each group) were enrolled. At 3 months follow-up, statistically significant differences for intermediate visual acuities were found between the three groups. Nonstatistically significant differences were observed for distance visual acuities and the changes in Catquest-9SF scores.

**Conclusion:**

Tecnis Eyhance™ provided better results in intermediate visual outcomes without adverse effects on patients’ quality of life.

## Introduction

Cataract extraction surgery is turning into a refractive surgery and will do so even more in future. Patients have become more demanding regarding visual quality and postoperative functional vision, wishing for total, or nearly total, independence from glasses. Monofocal intraocular lenses (IOLs) are still the most implanted lenses in the current scenario due, among other things, to the lower rate of optical side effects such as photic phenomena (glare, halos) compared to multifocal IOLs, which result from the refractive or diffractive morphology of the optics. Monofocal IOLs have a single point of focus for far vision and thus do not provide the intermediate vision required for the majority of our daily activities, such as working on a computer or tablet, playing music or sports, looking at the dashboard of a car and walking on uneven surfaces [1–7].

In recent years, the number of IOLs providing good distance and intermediate vision has increased. One of the reasons for this is that the average age of the surgery is decreasing; while, the needs of cataract patients are increasing. Patients over sixty engage in increasing activities, often indoors, in dimly lit environments, and at intermediate distances [7]. Introduced in 2019, the Tecnis Eyhance™ (ICB00) (Johnson & Johnson Vision, Irvine, CA, USA) was the first of a new generation of enhanced monofocal IOLs that allow patients to have high-quality vision in both intermediate and far distances when compared to standard aspherical monofocal IOLs. The ICB00 features refractive technology with a design that is free of rings. Its power, designed to keep the benefits of a monofocal IOL, gradually increases from the periphery to the center, creating a unique high-order aspheric profile on the anterior surface [7–10].

The present study aimed to compare the visual outcomes of a new-generation enhanced monofocal IOL with those of two commonly implanted monofocal IOLs in patients undergoing bilateral cataract extraction.

## Methods

### Patients and study design

A prospective, single-center, single-masked, randomized controlled clinical trial compared the bilateral implantation of two monofocal IOLs and one enhanced monofocal IOL. Patients scheduled for consecutive bilateral cataract extraction at the Trieste University Eye Clinic’s Cataract Surgery service were enrolled between November 2019 and May 2022. The enrolled patients were divided into three groups of 30 patients each, each of whom was a candidate for bilateral implantation of one of the three lenses under investigation: Tecnis Eyhance™ -ICB00 (Johnson & Johnson Vision), Tecnis^®^ 1-piece monofocal (PCB00) (Johnson & Johnson Vision) or Clareon^®^ monofocal (CNA0T0) (Alcon Laboratories). The second eye was implanted 1 month after the first.

Exclusion criteria included anterior segment pathology that could have a significant impact on outcomes (e.g., chronic uveitis, iritis, corneal dystrophy, keratoconus), axial length ≤ 21 mm or ≥ 26 mm, corneal topographic astigmatism higher than 0.75 D, diabetic retinopathy, uncontrolled glaucoma and or IOP > 24 mmHg, all kind of infections (acute ocular disease, external/internal infection, systemic infection), traumatic cataract, pseudoexfoliation syndrome, pupillary abnormalities including aniridia and/or pupillary diameter in mesopic conditions in distance vision ≤ 2.5 mm and ≥ 6 mm, microphthalmia, amblyopia, degenerative visual disorders (e.g., macular degeneration, optic nerve atrophy or retinal disorders), previous intraocular and corneal surgery, systemic or ocular pharmacotherapy which could impact the visual acuity and/or cause floppy iris syndrome and/or insufficient dilation according to the investigator's opinion, strabismus, nystagmus, pregnancy or lactation period for female patients.

Following the Helsinki Declaration, each patient provided written informed consent before participating in the study. The Hospital Ethics Committee approved the study (RCTICB00; protocol 202/2019). The trial was retrospectively registered (ClinicalTrials.gov Identifier: NCT06118944). Patients were consecutively randomized by block randomization and assigned in a 1:1:1 allocation ratio to three study arms using Sealed Envelope Ltd. 2017 [Available from: https://www.sealedenvelope.com/simple-randomiser/v1/lists]. An automatically generated unique randomization code was assigned to the randomized patient, followed by the abbreviation LE or RE to distinguish between left and right eyes, respectively.

### Assessments

All patients underwent a comprehensive preoperative ophthalmological examination before surgery, which comprised a complete medical history recording, including demographic data and systemic diseases, the measurement of monocular and binocular uncorrected distance visual acuity (UDVA) (at 4 m) and corrected distance visual acuity (CDVA), anterior segment slit lamp examination, Goldmann applanation tonometry, fundus oculi examination after dilatation with Tropicamide 1% eye drops, macular optical coherence tomography (Spectralis HRA + OCT; Heidelberg Engineering, Heidelberg, Germany), bilateral optical biometry (IOLMaster^®^ 700; Carl Zeiss Meditec AG, Jena, Germany, OPD), corneal topography and pupillometry (Scheimpflug camera—Sirius; C.S.O.) and quality of life assessment using Catquest-9SF questionnaire. Data acquired with IOLMaster^®^ 700 were used to calculate the IOLs' dioptric power. The IOL powers were calculated using the Barrett Universal II formula. The dioptric power with the expected refractive target closest to emmetropia was chosen.

Postoperatively, patients were evaluated 12 weeks (3 months) after the second eye surgery. Each patient underwent biomicroscopy of the anterior segment to assess the overall state of the operated eye and, subsequently, monocular and binocular visual outcomes with uncorrected and corrected visual acuity for far distance (UDVA, CDVA), uncorrected visual acuity for intermediate distance (UIVA), distance-corrected intermediate visual acuity (DCIVA), distance-corrected near visual acuity (DCNVA), corrected near visual acuity (CNVA). Near vision correction (NVC) spherical equivalent (SE) was recorded as well. Corrected visual acuity was obtained using the “Maximum plus” (or minimum minus) technique, a subjective refraction technique that produces the best vision with the minimum minus or maximum plus correction.

Early Treatment Diabetic Retinopathy Study (ETDRS) acuity charts were used to measure pre- and postoperative visual acuities. Distance Visual Acuity (DVA) was obtained with a 4 m ETDRS board illumination cabinet at high contrast (96%) with an 85 cd/m^2^ lamp filter tube (Precision Vision), intermediate visual acuity (IVA) was obtained with a 70 cm ETDRS printed chart (Precision Vision), and near visual acuity (NVA) was obtained with a 40 cm ETDRS printed chart (Precision Vision). Binocular corrected distance defocus curves were obtained. To produce defocus, a progression of IOLs in − 0.50 D increments was consecutively added (range + 2.00 to − 4.00 D), after which visual acuity was tested with 100% contrast ETDRS distance acuity charts at a test distance of 4 m. The self-administered validated Catquest-9SF questionnaire assessed patients' satisfaction with visual outcomes for daily life activities at 12 weeks.

### Intraocular lenses

ICB00 is a 1-piece acrylic aspheric refractive foldable posterior chamber IOL designed for placement in the capsular bag. This IOL is made of the same hydrophobic Sensar acrylic material and has the same overall geometry/dimensions (13 mm overall length and 6.0 mm optic diameter) as the standard 1-piece monofocal IOL. It has the same features as the PCB00 IOL, except for the modified aspheric anterior surface of the optic. The enhanced and standard monofocal IOLs are based on refractive technology without diffractive rings or zones and have the same IOL constant. The enhanced monofocal IOL has a refractive optical design with a higher-order aspheric anterior surface that creates a continuous power profile (the power increases continuously from the periphery to the center of the lens), which is intended to extend the depth of focus, thus improving vision for intermediate tasks compared with a standard monofocal IOL [7–10].

Moreover, ICB00 IOL compensates for corneal spherical aberration similarly to the standard 1-piece monofocal PCB00 IOL, adding a negative spherical aberration of 0.27 μm. The standard IOL PCB00 is a 1-piece UV-light filtering acrylic monofocal IOL with a modified prolate (aspheric) design on the anterior optic surface to reduce the overall spherical aberration to near zero once it is implanted. The CNA0T0 monofocal IOL is a one-piece aspheric, hydrophobic, monofocal non-toric IOL made of ultra-violet and blue-light filtering acrylate/methacrylate copolymer. It has the same mechanical design as the AcrySof^®^ model SN60WF with a reported 0.20 μm negative spherical aberration [11]. CNA0T0 IOL features include Stableforce^®^ modified-L haptics (Alcon Vision), a full 6.0-mm optic diameter, a proprietary square-edged design, and a 13.0-mm overall length.

### Surgery

Patients considered eligible during the preoperative visit were scheduled for surgery and were operated on by an expert surgeon (DT). Standard phacoemulsification through a 2.4 mm clear corneal incision followed by IOL insertion in the capsular bag is the routine approach for cataract extraction using topical anesthesia at our department (unless complicated cases). Anterior capsulotomies were performed as a continuous, curvilinear capsulorhexis of 5.0 to 5.5 mm diameter performed by manual capsulorhexis.

### Outcome measures

The study's primary goal was to evaluate and compare the clinical outcomes of three parallel groups of patients following bilateral implantation of the ICB00 IOL, PCB00 IOL, or CNA0T0 IOL. Considering the modified optical profile of the ICB00, the primary outcome was the evaluation of intermediate-distance visual performance. The primary endpoint was to compare groups in terms of binocular DCIVA at 12 weeks after the second eye implant. Secondary endpoints included monocular and binocular UIVA, UDVA, CDVA, DCNVA, CNVA, monocular NVC SE, binocular defocus curves and Catquest-9SF questionnaire scores.

### Statistical analysis

One-way ANOVA with partial eta squared (*η*_p_^2^) as an effect size indicator was applied to compare the groups regarding clinical and demographical variables. The between-group differences in postoperative data were evaluated with ANCOVA, including age-controlled covariate. In this case, all between-group comparisons performed with controlled covariates were carried out individually for each dependent variable. The Catquest-9SF data were fit to the Rasch model using WINSTEPS version 4.2 using the Andrich version of Rasch model estimates based on joint maximum likelihood estimation. A Rasch analysis compares the difficulty required to complete a task mentioned in the items to the participant’s ability level to perform that activity. Both the object’s difficulty and the individual’s ability are sorted on the same linear scale. If the data fit the Rasch model’s assumptions, the ordinal raw score is transformed into a valid Rasch scale. This scale is linear and has a logit unit, which is the natural logarithm of the odds ratio. Because the Catquest-9SF should be valid for measuring both before and postoperative patient data, Rasch analysis was done with preoperative and postoperative data stacked as a single dataset. The Bonferroni correction was applied to establish a statistical significance threshold resulting from multiple tests of other controlled covariates. After determining the distributional parameters significantly differentiating the groups, correlations between these parameters were calculated separately.

## Results

Clinical and demographical data of the three groups are reported in Table 1. After the ANOVA analysis, only age significantly differed between all three groups. The main difference in the age criterion was observed between the ICB00 and CNA0T0 (*p* = 0.001) groups.

**Table 1:** Population data analysis for PCB00, ICB00, CNA0T0

	PCB00 (Mean ± SD)	ICB00 (Mean ± SD)	CNA0T0 (Mean ± SD)	ANOVA	
				*p*-value	$\eta_{{\text{p}}}^{2}$
Age (y)	76.97 ± 6.1	75.73 ± 10.26	83.83 ± 7.28	0.001*	0.192
IOL powers (D)	22.32 ± 1.94	22.16 ± 1.79	21.91 ± 1.96	0.496	0.007
Pre-operative VA (logMAR)	0.31 ± 0.11	0.32 ± 0.12	0.33 ± 0.12	0.611	0.005
WTW (mm)	11.9 ± 0.34	11.09 ± 0.35	11.98 ± 0.35	0.369	0.008
ACD (mm)	3.03 ± 0.48	3.15 ± 0.39	2.94 ± 0.45	0.051	0.031
AL (mm)	23.3 ± 0.88	23.15 ± 0.61	23.41 ± 0.81	0.184	0.018
Scotopic pupil diameter (mm)	4.31 ± 0.72	4.25 ± 0.71	4.1 ± 0.64	0.284	0.014
Mesopic pupil diameter (mm)	3.57 ± 0.58	3.61 ± 0.61	3.51 ± 0.58	0.607	0.005
Photopic pupil diameter (mm)	2.82 ± 0.45	2.88 ± 0.5	2.8 ± 0.48	0.589	0.006

*ACD* anterior chamber depth, *AL* axial length, *ANOVA* analysis of variance, *D* diopters, *IOL* intraocular lens, $\eta_{{\text{p}}}^{2}$ partial etha squared, *SD* standard deviation, *WTW* white to white, *y* years

*Post hoc PCB00 versus ICB00: *p* = 1; PCB00 versus CNA0T0: *p* = 0.163; ICB00 versus CNA0T0: *p* = 0.00

Monocular and binocular visual acuities mean values and comparisons among groups are reported in Table 2.

**Table 2:** Postoperative visual acuities and refraction analysis for PCB00, ICB00, CNA0T0

	PCB00	ICB00	CNA0T0	ANCOVA with Age as a Covariate		
				*p*-value	$\eta_{{\text{p}}}^{2}$	Bonferroni Post hoc analysis
*Monocular*						
Mean SE ± SD	− 0.18 ± 0.41	0.03 ± 0.31	− 0.03 ± 0.33	0.004	0.059	PCB00vsICB00: *p* = 0.004; PCB00vsCLAREON: *p* = 0.075; ICB00vsCLAREON: *p* = 1
Mean UDVA ± SD	0.03 ± 0.08	0.003 ± 0.09	0.003 ± 0.07	0.141	0.022	
Mean CDVA ± SD	− 0.03 ± 0.04	− 0.03 ± 0.05	− 0.01 ± 0.04	0.198	0.018	
Mean UIVA ± SD	0.32 ± 0.1	0.2 ± 0.13	0.34 ± 0.1	< 0.001	0.224	PCB00vsICB00: *p* < 0.001; PCB00vsCNA0T0: *p* = 1; ICB00vsCNA0T0: *p* < 0.001
Mean DCIVA ± SD	0.29 ± 0.11	0.17 ± 0.13	0.31 ± 0.11	< 0.001	0.187	PCB00vsICB00: *p* < 0.001; PCB00vsCNA0T0: *p* = 0.921; ICB00vsCNA0T0: *p* < 0.001
Mean DCNVA ± SD	0.38 ± 0.11	0.25 ± 0.13	0.36 ± 0.12	< 0.001	0.178	PCB00vsICB00: *p* < 0.001; PCB00vsCNA0T0: *p* = 0.885; ICB00vsCNA0T0: *p* < 0.001
Mean CNVA ± SD	0.05 ± 0.07	0.02 ± 0.06	0.04 ± 0.06	0.076	0.028	
Mean NVC SE (diopter) ± SD	2.07 ± 0.52	1.4 ± 0.6	2.22 ± 0.68	< 0.001	0.261	PCB00vsICB00: *p* < 0.001; PCB00vsCNA0T0: *p* = 0.569; ICB00vsCNA0T0: *p* < 0.001
*Binocular*						
Mean UDVA ± SD	− 0.05 ± 0.06	− 0.03 ± 0.07	− 0.03 ± 0.06	0.259	0.031	
Mean CDVA ± SD	− 0.08 ± 0.04	− 0.07 ± 0.05	− 0.07 ± 0.04	0.812	0.004	
Mean UIVA ± SD	0.32 ± 0.11	0.17 ± 0.12	0.31 ± 0.09	< 0.001	0.285	PCB00vsICB00: *p* < 0.001; PCB00vsCNA0T0: *p* = 0.969; ICB00vsCNA0T0: *p* < 0.001
Mean DCIVA ± SD	0.29 ± 0.01	0.13 ± 0.11	0.29 ± 0.09	< 0.001	0.327	PCB00vsICB00: *p* < 0.001; PCB00vsCNA0T0: *p* = 1; ICB00vsCNA0T0: *p* < 0.001
Mean DCNVA ± SD	0.36 ± 0.11	0.23 ± 0.11	0.33 ± 0.12	< 0.001	0.212	PCB00vsICB00: *p* < 0.001; PCB00vsCNA0T0: *p* = 0.999; ICB00vsCNA0T0: *p* < 0.001
Mean CNVA ± SD	0.02 ± 0.04	0.02 ± 0.05	0.01 ± 0.02	0.282	0.028	

*ANCOVA* analysis of covariance, *CDVA* corrected distance visual acuity, *CNVA* corrected near visual acuity, *DCIVA* distance-corrected intermediate visual acuity, *DCNVA* distance-corrected near visual acuity, *NVC* near vision correction, $\eta_{{\text{p}}}^{2}$ partial etha squared, *SD* standard deviation, *SE* spherical equivalent, *UDVA* uncorrected distance visual acuity, *UIVA* uncorrected intermediate visual acuity

**Fig. 1:**
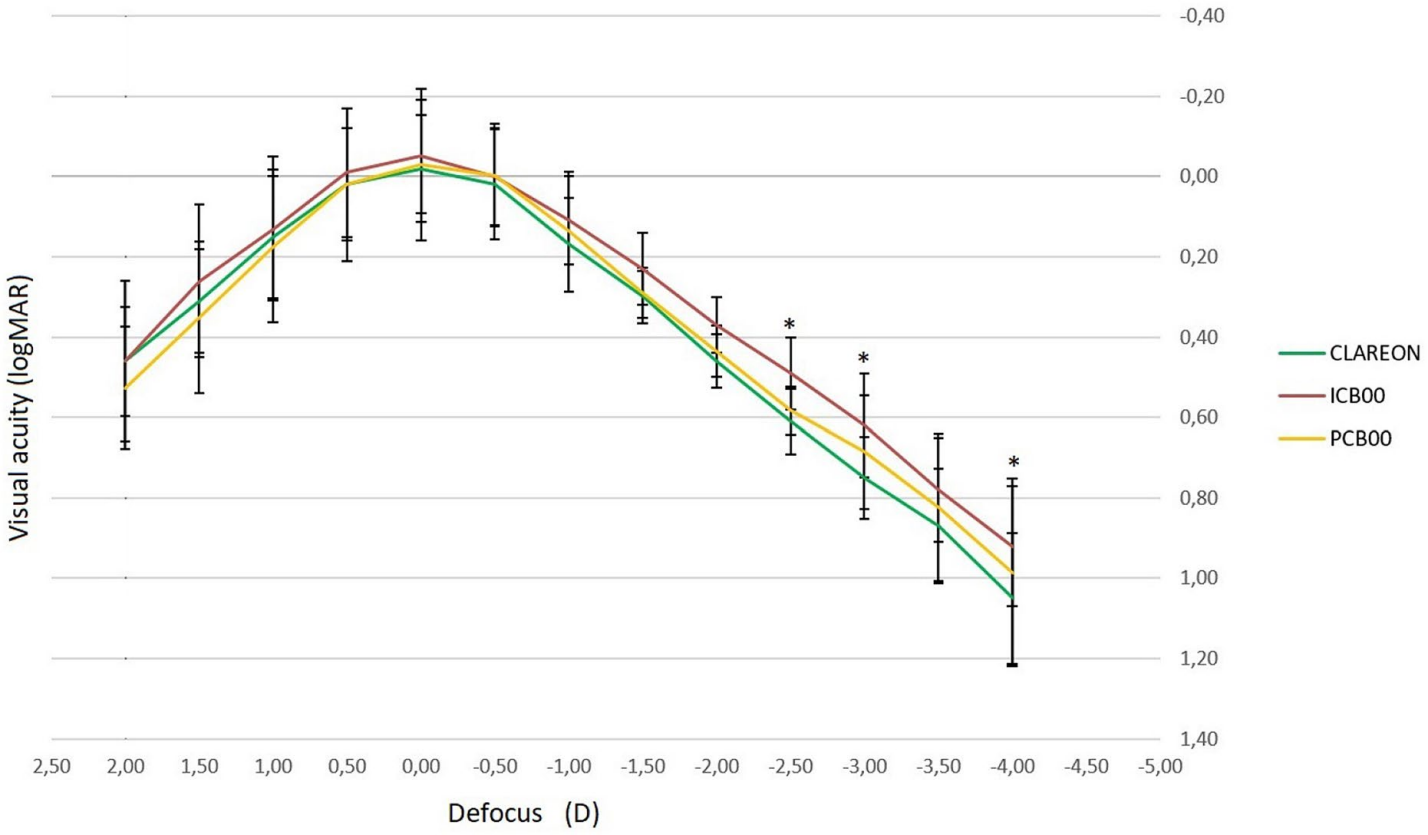
Mean binocular defocus curves for the intraocular lens groups (ICB00, PCB00, CNA0T0) across defocus levels (+ 2 D to − 4 D). Thirty patients (60 eyes) per group were analyzed

We observed a statistically significant difference among groups for monocular and binocular intermediate vision and DCNVA. Moreover, we found a statistically significant lower value of the mean NVC SE for the ICB00 group. After the ANCOVA analysis with age covariate, only the mean values of postoperative results for the ICB00 group were significantly different (*p* < 0.001) compared to the other two groups. There were no statistically significant differences among the three IOL groups between Catquest-9SF pre and postoperative results. Mean binocular defocus curves are shown in Fig. 1.

## Discussion

As the ESCRS Functional Vision Working Group has recently noted, intermediate distance vision is essential for most common and practical daily tasks, such as using a computer or seeing the dashboard of a car. This raised awareness has led to the development of an increasing number of novel IOL designs, including multifocal, extended depth of focus (EDOF) and next-generation enhanced monofocal IOLs, that can restore vision at various distances [5, 12]. Since 2019, a new class of monofocal IOLs has been introduced to meet patients' demand for improved intermediate distance vision while minimizing risks and costs. We compared visual outcomes and patient satisfaction following the bilateral implantation of one of two high-quality aspheric monofocal IOLs (PCB00 or CNA0T0) versus the bilateral implantation of the first of the new-generation monofocal IOLs (ICB00). In our study, the refractive outcomes for distance were excellent for all IOL groups, as previously observed by other studies [7, 10, 13–20]. We found a postoperative subjective SE close to 0 and similar between groups, with slightly higher variability in the PCB00 group. However, the mean values were not significantly different since different pupil sizes alone could result in different depths of focus; without considering the type of the implanted IOL, pupil size measurements before VA testing were taken. In mesopic conditions where visual acuity tests were done, pupil size was not significantly different between the three groups. The enhanced monofocal IOL outperformed the conventional monofocal IOLs regarding intermediate vision performance (monocular and binocular).

Similarly, Auffarth et al., comparing the enhanced monofocal IOL and the standard monofocal IOL, reported similar visual outcomes to the results of this study, with significant improvements in intermediate vision achieved by the enhanced monofocal IOL. Auffarth et al. demonstrated the enhanced monofocal IOL to significantly improve intermediate vision (monocular and binocular DCIVA and UIVA) by at least 1-line versus the standard monofocal IOL [21]. The standard arm length (60–70 cm) is the preferred screen-viewing distance for laptops and tablet devices, so we chose 70 cm as the intermediate distance. Moreover, this distance has already been used in other publications, which aids in comparison with studies performed with other IOLs [19, 22]. Corbelli et al. compared the ICB00 with the same brand, EDOF ZXR00. The authors found that at the − 1.0 D defocus level, simulating vision at a distance of 1 m, the visual acuity achieved by the ICB00 was not statistically significantly inferior to the ZXR00. This result highlights the non-inferiority of the enhanced monofocal ICB00 in intermediate-distance performance compared with the classic ZXR00 [17]. Nanavaty et al. compared ICB00 with conventional monofocal aspheric RayOne IOL (Rayner, UK). They found that Eyhance IOL provided better DCIVA and broader defocus curves than the RayOne IOL. There was no difference in CDVA or patient-reported outcomes. We did not study corneal aberrations in our study, but Nanavaty et al. found that although there were some differences in aberrations when measured with normal pupil size, they were not clinically significant [23].

Yangzes et al. reported that monocular UNVA was better (*p* < 0.01) for the ICB00 group (0.43 ± 0.13 logMAR) than for the PCB00 group (0.61 ± 0.16 logMAR) [7]. In our study, monocular UNVA (0.25 ± 0.13 logMAR) and binocular UNVA in the ICB00 group (0.23 ± 11 logMAR) were statistically significantly better than the other two groups (*p* < 0.001) [7]. Interestingly, Lee et al., comparing the ICB00 and ZXR00 IOLs, found binocular UNVA was comparable, though spectacle independence was higher in the ZXR00 group, at the expense of increased glare and halos [18]. Moreover, in our sample, the NVC (diopters) in the ICB00 group was significantly lower than in the other two groups. Defocus curves are widely used to objectively measure the performance of an IOL at various distances [7, 24]. In our work, the ICB00 group showed a significantly better visual acuity across defocus levels of − 1.50 to − 4.00 D, as observed by other authors, with good intermediate distance vision [7, 10].

We employed the Catquest-9SF [25–28] to assess patient-reported outcomes. Despite the higher visual acuity in the ICB00 group for UIVA, DCIVA and DCNVA, we found no statistically significant differences in Catquest-9SF scores between groups based on Rasch analysis. We hypothesized that this could be explained by the fact that the questionnaire specifies performing the tasks with correction, thereby reducing differences in those cases where glasses for intermediate and/or near vision were worn. A new questionnaire explicitly designed for new-generation IOLs and an extended range of vision IOLs should be advocated.

It is remarkable to underline that our study was prospective and randomized. Moreover, to our knowledge, no other scientific work in the literature compares the ICB00 IOL performance to two groups of standard monofocal IOLs (the PCB00 and the CNA0T0). In our study, we observed a statistically significant difference in the age of the three groups, with older patients in the CNA0T0 group. Moreover, the study has not included an analysis of higher-order corneal aberrations. These two factors should be considered when analyzing the results and might represent a limitation of the current study. Another limitation might be the lack of objective contrast sensitivity and visual quality tests. However, a recent meta-analysis on enhanced monofocal IOLs has reported no increased risk of contrast sensitivity loss or increased incidence of photic phenomena [29].

In conclusion, compared to standard monofocal IOLs, the Tecnis Eyhance™ IOL significantly enhanced unaided intermediate vision while maintaining comparable distance performance. Furthermore, in this preliminary study, we only included patients who did not have ocular comorbidities. Future studies would be useful to assess whether the performance of the ICB00 IOL is comparable to that of a monofocal IOL in patients with ocular comorbidities.
